# Using adult learning characteristics and the humanities to teach undergraduate healthcare students about social determinants of health

**DOI:** 10.1057/s41599-023-01599-w

**Published:** 2023-03-18

**Authors:** Elizabeth A. Brown, Hannah Kinder, Garrett Stang, Wendy Shumpert

**Affiliations:** 1 Old Dominion University, Norfolk, VA USA; 2 Medical University of South Carolina (MUSC), Charleston, SC USA

**Keywords:** Health humanities, Medical humanities

## Abstract

Authors used an andragogy framework to help undergraduate allied health students better understand social determinants of health (SDOH) using a photo essay assignment. The study examined students’ perceptions of SDOH in various communities, description of health outcomes associated with their chosen SDOH, and lessons learned and suggestions to improve the assignment for future cohorts. Data were extracted from photo essays from 2019–2021 and entered in Microsoft Excel and Word for data analysis after course completion. Conventional qualitative content analysis was used to analyze student evaluation data from open-ended questions. Data were extracted from 53 student essays from 2019 to 2021. Most photo essays described communities in South Carolina (*n* = 42, 79.2%), urban areas (*n* = 37, 69.8%), or intermediary SDOH (75.5%). Several themes emerged concerning lessons learned (awareness and empathy are key to addressing SDOH), health equity (collaboration is necessary to provide resources, especially for underserved populations), and constructive feedback for the instructor (more time to discuss SDOH and assignment with peers and instructor). Faculty must work with students to think about more upstream factors like policy and cultural and societal values. Collecting evaluation data, specifically lessons learned and constructive feedback for faculty, can help faculty continuously improve course topics and assignments. Following a transparency framework can support student success and help faculty become effective leaders in the classroom while teaching subjects like SDOH and social justice.

## Introduction

Social determinants of health (SDOH) refer to the factors that influence one’s quality of life and various health outcomes like morbidity, mortality, life expectancy, healthcare expenditures, and much more (Artiga & Hinton, 2018). These factors may also be viewed as predictors of various health outcomes and include access to healthcare, education, socioeconomic status, and physical conditions such as living environments (Baverstock et al., 2018). Healthcare leaders are recognizing the growing significance of the impact of SDOH on health and health equity. Educators in medicine are using classroom space to teach about SDOH and their effect (Doobay-Persaud et al., 2019; Klein et al., 2011; Martinez et al., 2015; Sharma et al., 2018; Witten & Maskarinec, 2015); however, not much is seen in the literature for allied healthcare professionals (Brown et al., 2021).

Authors employed several lenses to teach SDOH to allied healthcare professionals and students at a large academic medical center in the southeastern United States: (1) characteristics of adult learning, (2) aspects of a transparency framework, and (3) fundamentals of the humanities.

### Adult learning

Adult learning prompts students to (a) use their previous life experiences and critical self-reflection to continue learning (experiential learning or project-based learning), (b) think differently about their world (transformative learning), and (c) take responsibility for their learning and connect assignments to current profession (self-directed learning) (Western Governors University, 2020).

### Transparency framework

The Transparency in Learning and Teaching Project (TILT) Framework reinforces adult learning by helping students understand why they should complete the assignment. For example, the TILT Transparency Framework suggests providing students with the purpose of the assignment, skills and knowledge gained from completing the assignment, and a clear task list to complete the assignment (Winkelmes, 2014). The TILT Transparency Framework provides the instructor the opportunity to encourage adult learners to establish the relationship between the course assignment and their personal or professional lives.

### Humanities

The humanities, also called the medical humanities, were used so students may have a creative space to capture SDOH in various communities using photography and discuss SDOH impact on health outcomes. According to Wald et al. (2019) medical humanities incorporate aspects of the human experience and may be used to help increase students’ reflection and awareness, professionalism, and the quality of life of both patient and healthcare professional.

Healthcare professionals, particularly allied health providers, can benefit from learning about SDOH through the humanities and apply their learning in practice with diverse patients. SDOH are complex issues that tend to be overlooked in healthcare; however, classroom space can be used to promote discussions about SDOH, disease prevention, and health equity. If implemented correctly in healthcare curricula, incorporating medical humanities while teaching about SDOH could improve students’ understanding and patients’ experiences in the healthcare setting.

One approach that combines medical humanities and SDOH is a photography essay. A photography essay is a tool that is used to help tell a story. Through photo essays, individuals can capture a photo that they believe to be representative of SDOH and explain their impact on various health outcomes. In one study with first-year nursing and pharmacy students, an instructor used photography to assess students’ understanding of SDOH in their communities (Baverstock et al., 2018). Students were instructed to take a photograph in their community, link it to a SDOH, and provide a solution to addressing SDOH depicted in their photograph. The study found two emerging themes from student data–importance of education and the role of the government–and reported student challenges (Baverstock et al., 2018). The current study hopes to add to the previous study by (1) including a range of allied health professionals in the data analysis, (2) using a SDOH Framework to identify and categorize SDOH and overlay students’ chosen SDOH over the SDOH Framework, and (3) sharing the project guidelines, developed using the TILT Transparency Framework, and grading rubric so that other instructors may build upon this work with different student groups.

### Study purpose

The purpose of this study was to assess (1) adult learners’ understanding of SDOH in a community setting and how students, who are allied health professionals, interpret the impact of SDOH on health and (2) learn approaches to improve the photo essay assignment for other faculty in academia.

## Methods

### Program description

The SDOH course is a required course for students at one large academic health center in the southeastern United States. Generally, students are allied health professionals and work full-time as Occupational Therapy Assistants, Physical Therapy Assistants, Dental Hygienists, Radiological Technologists, Respiratory Therapists, Certified Nursing Assistants, Certified Medical Assistants, or Emergency Medical Technicians.

### Course description

The SDOH Photography Essay is a midterm paper required in the SDOH course offered at an academic health center (Brown et al., 2021; Gregory et al. (2022)). The SDOH course uses the World Health Organization’s SDOH Conceptual Framework to examine and discuss the fundamental determinants of health, including socioeconomic status and social gradient, early childhood development, neighborhoods and communities, behaviors, biological factors, psychosocial factors, immigration status, social exclusion and social support, healthcare system, and health policy (World Health Organization, 2010). Further, the course dedicates several weeks to topics like ethical principles and cultural awareness.

#### Photo essay assignment

While the SDOH course incorporates various assignments like a Modified Privilege Walk (MPW) (Brown & White, 2020; Gregory et al., (2022); Witten & Maskarinec, 2015), student-developed case study (Cantey et al., 2017), service-learning experience (Taylor et al., 2017), or perspective (Brown, 2020) to promote discussions about SDOH, privilege, and health equity (Brown et al., 2021), this paper will focus on a photo essay assignment, which serves as the course midterm paper. The instructor provided students with project guidelines for the Photography Essay assignment and a grading rubric so students may evaluate their work before submission (see supplemental material). The project guidelines for the Photography Essay assignment followed the TILT Framework, which supports classroom equity and student success by clearly detailing the following items: assignment due date, assignment purpose, skills and knowledge gained, and a task list (Winkelmes, 2014). Students were required to take at least one photo of a SDOH in a physical community setting and write a four-to-five-page paper discussing SDOH (see supplemental material). Students provided a definition of SDOH, described the SDOH in their photographs, described community demographics from the physical community that they took the picture, and explained how the SDOH in the picture could impact at least two different health outcomes (e.g., mortality, morbidity, life expectancy, etc.). The first two cohorts (2019 and 2020) chose one health outcome. The third (and final cohort) in 2021 chose two health outcomes. Students also described how to improve health equity regarding their chosen SDOH and provided a “Lessons Learned” section where they also recommend approaches to improve the assignment for future cohorts. Students were instructed to place their photo in the Appendix of the paper.

### Research design

Authors used qualitative methodology to analyze data from the SDOH Photography Essay assignment. Qualitative methods focused on collecting and analyzing data to explore subjects’ experiences and understanding of various social or physical communities were used for this project (Tong et al., 2007). Open-ended responses from assignment questions were analyzed using an inductive approach, where the analysis was data driven and not particularly focused on a framework or theory. The inductive approach allowed flexibility where the research team could focus purely on students’ responses to open-ended questions to gather new knowledge.

Authors used conventional qualitative content analysis, which is an ideal approach in describing a phenomenon when limited research literature or theories are available (Erlingsson & Brysiewicz, 2017; Hsieh & Shannon, 2005). Content analysis allows for systematic and open coding to explore data and find patterns within the text (Vaismoradi et al., 2013). One of the advantages of conventional content analysis is researchers are not required to have pre-determined categories to impose on study participants’ data, allowing authors to describe students’ data in essays (Hsieh & Shannon, 2005).

### Participants

Participants included pre-health professional students in an online, undergraduate degree program at an academic medical center. Many students were “non-traditional” undergraduate students who work in allied health professions.

### Data collection

After the conclusion of the course and grades were submitted for each cohort, the instructor extracted relevant data from photo essay assignments and input data into a primary Excel and Word document. The primary Excel document included an identifier and student names so the instructor could retrieve additional information at a later date, if needed. However, a second Excel document with de-identified data was created for data analysis purposes. Open-ended responses were copied into a Word document for qualitative data analysis. The Word document with open-ended responses contained de-identified data.

#### Study variables

The following data were captured from each photo essay and added in Excel: Identifier, cohort year, city/town, county, rural county status, state, description of photo, chosen SDOH, structural vs. intermediary SDOH, and health outcome. Additional data concerning solutions to address health equity, lessons learned, and recommendations to improve the assignment were put in a Word document. Primary photos were saved in a PowerPoint file. Photos with an individual’s face were excluded and not saved in the PowerPoint file. Rural county was coded in the following manner: (0) Not a rural county or (1) Rural County. While there are various definitions for rural counties (Health Resources and Services Administration, 2018; Warren & Smalley, 2014), authors chose to identify rural counties as a county with less than 50,000 residents (Warren & Smalley, 2014). Structural SDOH was coded in a similar fashion—(0) Not a structural SDOH (intermediary SDOH) or (1) Structural SDOH—based on the WHO SDOH Conceptual Framework (World Health Organization, 2010).

#### Data analysis

The instructor on record (EAB) worked with one co-author (HK) to review de-identified data in an Excel document after the completion of all courses. Both authors checked the rural county status using the United States Census Bureau Quick Facts data to check population estimates from the April 2020 Census (United States Census Bureau, 2020). Both authors reviewed and discussed if SDOH were considered structural or intermediary based on the WHO SDOH Conceptual Framework. The students’ photos and photo description were used to help determine which SDOH were structural or intermediary. Where the two authors disagreed, a third party, who is a Ph.D trained health equity researcher, was asked to provide their thoughts to reach consensus.

Two members of the research team (EAB & HK) read students’ de-identified responses in a Word document for (1) How to improve health equity, (2) Lessons learned from completing the assignment, and (3) Constructive feedback to improve the assignment for future cohorts. Afterward, those same two members of the research team developed an initial draft codebook based on preliminary coding of the student data and met to discuss revisions of the codes and codebook. The final codebook was developed for each section–Health equity, Lessons learned, and Constructive feedback–then all transcripts were coded individually by both coders. Each time student data were coded, the coder wrote comments describing their thoughts about the student’s response. Comments were shared with each coder and discussed during virtual meetings from January 2022 to May 2022. When disagreements occurred, the two coders met to discuss their thoughts to reach consensus. Coders continuously met for several months to review the codebook and codes in the data until several themes emerged.

### Institutional review board/ethical considerations

The Medical University of South Carolina Institutional Review Board (IRB) determined this study to be quality improvement (QI)/program evaluation, not human subjects research, thus the project was not subject to further IRB review or approval. Additionally, students were (1) provided information on photography etiquette (e.g., being respectful of personal space), (2) asked to practice extreme safety in unsafe areas and neighborhoods (e.g., not talking to strangers or going in unfamiliar areas alone), and (3) charged with following all state and local policies related to the Coronavirus Pandemic (e.g., wearing a face mask).

## Results

Data were collected from 53 photo essay assignments from three separate cohorts from 2019 to 2021. There were 42 (79.2%) photo essays describing communities in South Carolina (SC), seven (13.2%) exploring communities outside of SC, and four (7.5%) that did not specifically identify the state where the photo was captured (Table 1). Photos were taken in other states, including Florida, North Carolina, Louisiana, West Virginia, Connecticut, New York, and Tennessee. The majority of photos were taken in urban areas (*n* = 37, 69.8%), while six photos (11.3%) were taken in rural communities. There were 10 (18.9%) photo essays that did not list the county name and were considered as missing data. Many students focused on community characteristics like housing, transportation, food insecurity and the built environment (*n* = 15, 28.3%), homelessness (*n* = 14, 26.4%), or access to medical, dental, or mental healthcare (*n* = 11, 20.8%). Other SDOH captured included socioeconomic status (*n* = 8, 15.1 %) and policy issues like gentrification and annexation (*n* = 2, 3.8%). A few students also focused on biology/genetics, smoking behavior, and social acceptance (*n* = 3, 5.7%).

**Table 1:** Student Photo Essay Characteristics, *n* = 53^1^.

	2019 *n* = 21	2020 *n* = 16	2021 *n* = 16	Total *n* = 53
State				
South Carolina	16 (76.2%)	13 (81.2%)	13 (81.2%)	42 (79.2%)
Other	3 (14.3%)	3 (18.8%)	1 (6.3%)	7 (13.2%)
Community				
Urban	15 (71.4%)	9 (56.3%)	13 (81.3%)	37 (69.8%)
Rural	3 (14.3%)	3 (18.8%)	0 (0.0%)	6 (11.3%)
SDOH				
Intermediary	18 (85.7%)	11 (68.8%)	11 (68.8%	40 (75.5%)
Structural	3 (14.3%)	5 (31.3%)	4 (25.0%)	12 (22.6%)

### Structural and intermediary SDOH

**Fig. 1:**
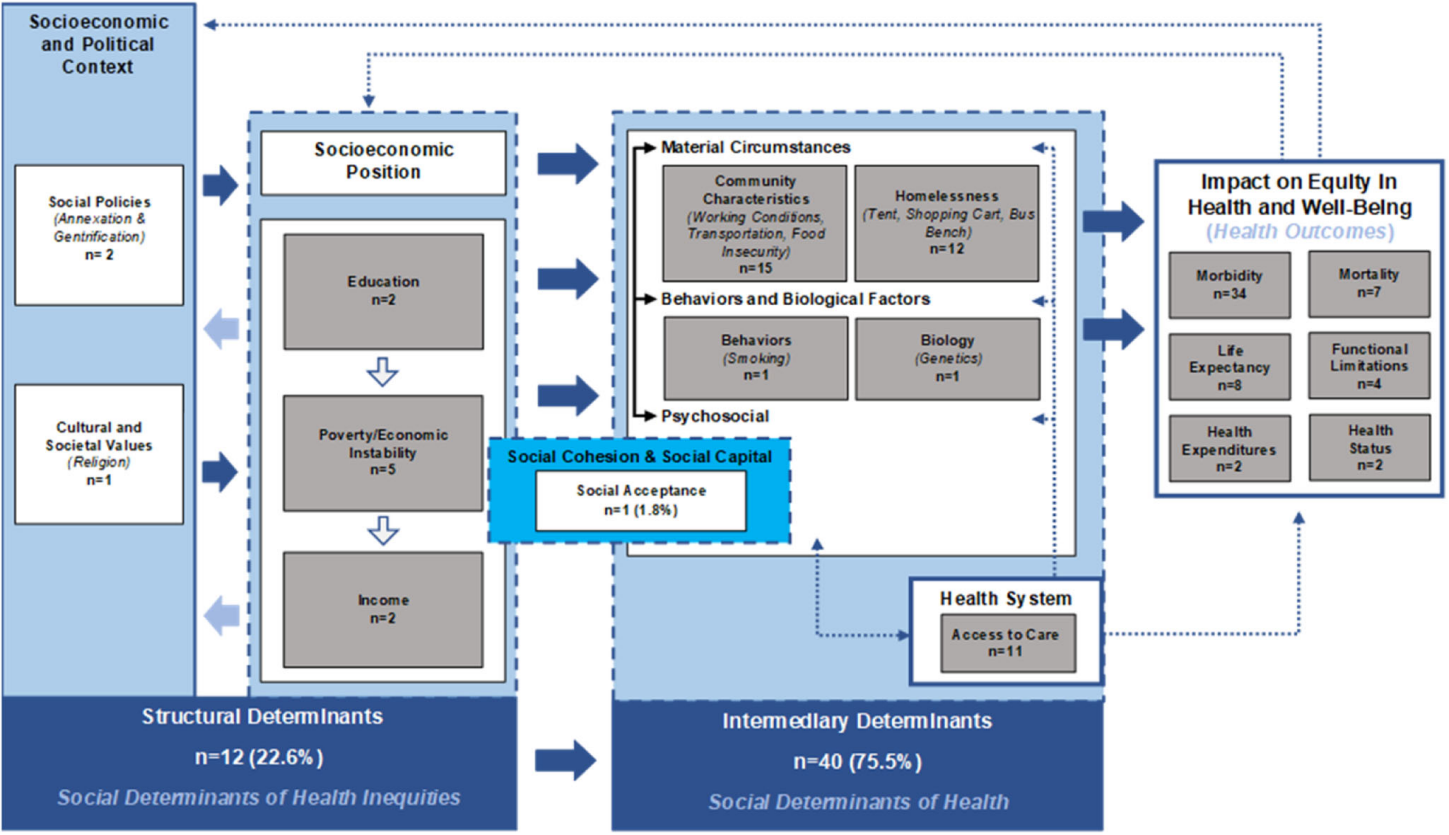
Depiction of student-identified SDOH mapped to an adapted Worth Health Organization’s (WHO) Commission on Social Determinants of Health (CSDH) conceptual framework.

**Fig. 2:**
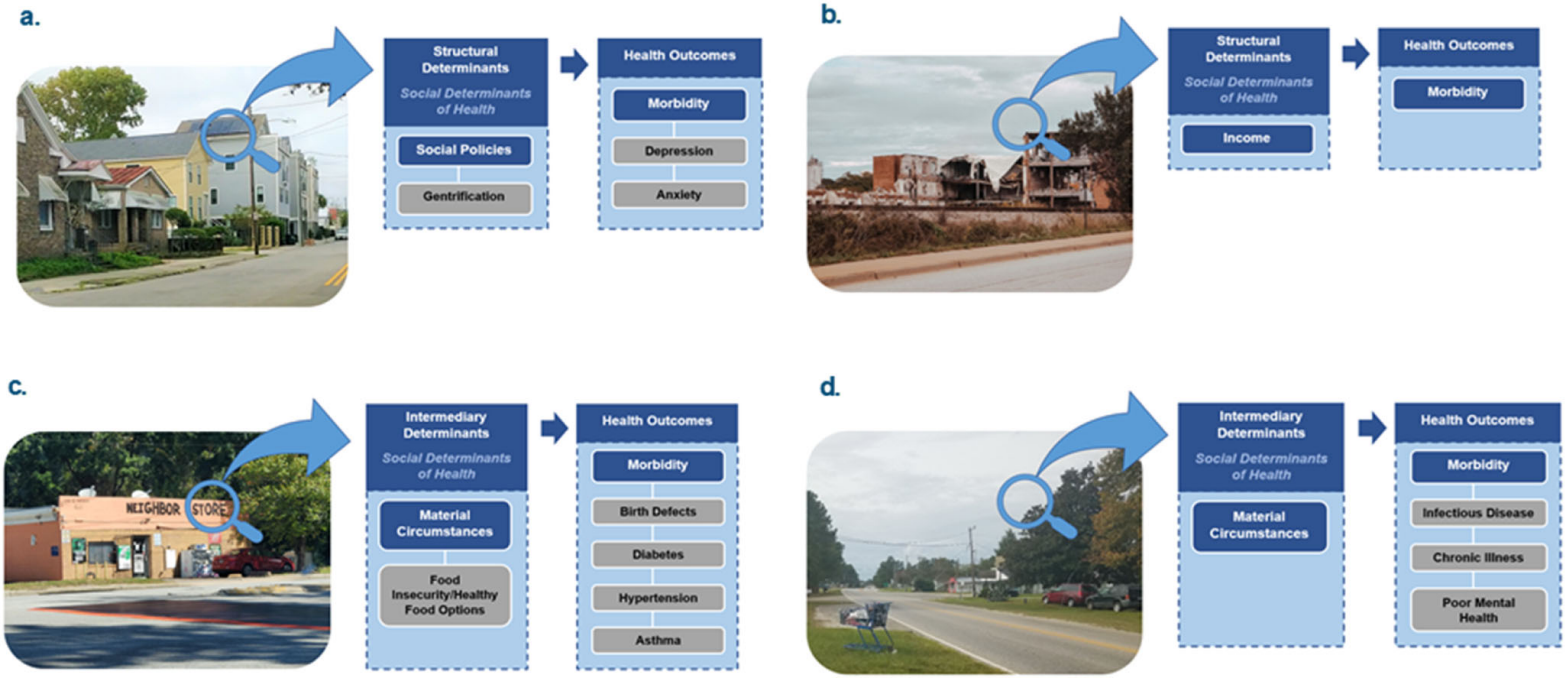
Students’ perceived structural and intermediary determinants of health and the link to various health outcomes. **a** Perceived structural determinants of a community from the adult learner’s perspective of new housing construction. **b** Perceived structural determinants of a community from the adult learner’s perspective of dilapidate building. **c** Perceived intermediary determinant of a community from the adult learner’s perspective of a local convenience store. **d** Perceived intermediary determinants of a community from the adult learner’s perspective of an abandoned shopping cart.

Based on the WHO SDOH Framework’s categorization of structural and intermediary SDOH, 40 students (75.5%) discussed intermediary SDOH like the healthcare system and access to care and material circumstances (e.g., reliable transportation, food insecurity, and homelessness) (Table 1) (Fig. 1). Twelve students (22.6%) discussed structural SDOH such as factors related to socioeconomic status or social policies. In 2019, one student (1.8%) described social acceptance, which could fall under both structural and intermediary in the WHO Framework. When describing SDOH, students were instructed to discuss which health outcome their chosen SDOH may directly impact and explain how. Most students focused on how SDOH impact morbidity (Table 2). For example, one student connected limited access to education with stress and anxiety. A second student discussed how one’s religion could positively impact morbidity (e.g., a person of the Mormon faith may abstain from smoking cigarettes and drinking alcohol). Another student chose to discuss how economic instability can impact life expectancy. Additional examples are provided in Fig. 2.

**Table 2:** Students’ Chosen Health Outcomes related to Social Determinants of Health^2^.

	2019 *n* = 21	2020 *n* = 16	2021 *n* = 16
Health outcomes			
Morbidity	10	12	12
Mortality	3	1	3
Life expectancy	4	3	1
Health status	1	1	0
Health expenditures	2	0	0
Functional limitations	3	0	1

### Lessons learned

Fifty-two students (98.1%) provided lessons learned. The codes that appeared the most regarding lessons learned were awareness (*n* = 43), impact (*n* = 17), and interconnectedness of SDOH (*n* = 17). The major theme recognized was that awareness and empathy are key to better understanding and addressing SDOH. Another theme focused on the impact and interconnectedness of SDOH throughout the life cycle. Many students reported that the assignment was “eye-opening,” especially researching and seeing data about poverty, mortality, and food insecurity. One student reported, “The assignment inspired me to volunteer with a nonprofit organization to help families in poverty.”

### Improving health equity

Forty-nine students (92.4%) responded with ways to improve health equity. The codes that appeared the most for improving health equity included specific recommendations to improve health equity (*n* = 37), vulnerable populations (*n* = 24), and access (*n* = 18). Healthcare access (*n* = 17), policy/program (*n* = 16), and awareness/education (*n* = 14) followed closely behind. The emerging theme was that organizations, including government agencies, need to collaborate to provide access to various resources, specifically for vulnerable populations like the low income and children. A second theme focused on how policies and programs, coupled with awareness and education, can improve healthcare access for vulnerable populations. In reference to improving health equity, one student stated, “Public policies helping families with special needs children, especially in rural areas. [We should] establish more satellite clinics in remote areas and offer more telehealth.” Another student emphasized the need to “support food banks, increase healthier food options (perishable foods) at food banks, and provide health education material at food banks.”

### Constructive feedback

Thirty-nine students (73.5%) provided suggestions to improve the assignment. The codes that appeared the most were advice to future students (*n* = 18) and advice to the instructor (*n* = 18). Codes regarding clarity (*n* = 8), successes (*n* = 7), and challenges (*n* = 3) were also captured. Advice to future students focused on choosing a SDOH they are familiar with, passionate about, or one that is related to their profession. Advice to the instructor included (1) splitting the paper into multiple assignments that include group discussions to promote conversations about SDOH, (2) submitting a draft of the paper for feedback and corrections before submitting the final assignment, and (3) offering one on one meetings to walk through the paper. Students used words and phrases like “straightforward,” “organized,” “clear and easy to follow” and “helpful to stay on track” when discussing the assignment guidelines and rubric. As for success, students called the assignment “a wonderful experience,” “well-rounded,” and “informative and educational.” There were a few challenges noted, including allocating more time into the curriculum to work on the project and being uncomfortable with discussing community demographics.

## Discussion

The SDOH Photo Essay assignment encouraged students to identify and describe SDOH in a physical community via photography. In addition, students had to explain how their SDOH was connected to a particular health outcome, suggest approaches to improve health equity, describe lessons learned from completing the assignment, and provide constructive criticism. The majority of students captured photos that depicted intermediary SDOH in primarily urban communities and connected SDOH to morbidity versus other health outcomes like life expectancy or mortality. Rich qualitative data led to themes from lessons learned and improving health equity. Students grasped the interconnectedness of complexity of SDOH and recognized that empathy and awareness are key to understanding SDOH. As for improving health equity, students felt that policies, programs, and collaborations among various organizations was key to addressing SDOH in vulnerable populations, specifically the low income and children. Students provided advice to future cohorts as well as constructive criticism for faculty who may be interested in implementing a similar assignment in the classroom.

### Structural vs. intermediary SDOH

Intermediary SDOH, such as material circumstances (e.g., living and working conditions, food availability, healthcare system, etc.), were the most common SDOH captured in photos and discussed in papers. Admittedly, it may be more difficult to capture structural SDOH, such as policies, societal values, and racism, in a photograph. We should note that when students discussed ways to improve health equity, policy and programs came up several times, illustrating students’ understanding that changes to upstream factors are needed to influence factors downstream like healthcare access, homelessness, and access to education. According to Murray (2021) faculty should help students make the connection between SDOH and health by placing social justice at the core of teaching SDOH and focusing on upstream factors like policy. Another approach to focus on structural SDOH is to add more community engagement activities. Khazanchi et al. (2021) included a community engagement component in their evaluation to highlight structural SDOH and their impact in communities. Authors discuss “community-engaged pedagogy” where authors focused on inequities in terms of social, political, and historical contexts to discuss inequities with students, public health practitioners, and community leaders (Khazanchi et al., 2021). During the course evaluation, authors found that community engagement was listed as a strength of the curriculum, and students requested more student engagement and small group discussions (Khazanchi et al., 2021). In our study, morbidity was the most prevalent health outcome discussed in papers, which is to be expected as we continuously see how SDOH like eating habits, physical activity, access to fresh food can impact the likelihood of chronic diseases like diabetes and hypertension.

### SDOH and health equity impact

As faculty create assignments about SDOH or social justice, we must push students to go outside of their comfort level and discuss upstream factors and focus on next steps like describing approaches to advancing health equity. Faculty interested in recreating a SDOH photo essay may have students (1) only focus on structural SDOH in their photos and (2) require students to discuss health outcomes other than morbidity. Are there creative ways in photography to capture more upstream factors and discuss their impact on health outcomes and health disparities in various physical and social communities? One author describes a structural competence framework where interprofessional teams study and discuss how structures create and sustain racial inequity that impact health (Neff et al., 2020). Authors described how health professionals can increase empathy by shifting blame away from the patient and placing it on systems of inequities (Neff et al., 2020). While a majority of students did not take a photo of a structural SDOH, students in the 2020 and 2021 cohorts had the opportunity to explore structural-level SDOH in their final assignment where they view a documentary titled “13th” and write a reflection paper explaining how public policy may impact psychosocial factors and assess if public policies related to mass incarceration are equitable. Before the COVID-pandemic in 2020, students were required to volunteer with a nonprofit organization and write a SDOH reflection paper about their experience for the final paper, and, in course evaluation data, students suggested adding more volunteer hours for the final SDOH reflection paper (Brown et al., 2021). Additionally, HCS students take a Health Policy course where they are required to write a health policy analysis, researching key stakeholders, formulating policy options with advantages and disadvantages, and discussing the impact of their policy option on various social communities. Therefore, while many students did not capture structural determinants in their photos, there are other opportunities in their educational program to examine and discuss various policies as structural determinants.

### Students’ lessons learned

According to student data about lessons learned, students felt more aware concerning SDOH and their impact and expressed empathy, especially for vulnerable populations. Completing assignments like the photography essay may bolster students’ sense of awareness about SDOH and empathy for underprivileged or underserved communities. Before addressing any problem, an individual must have some sense of awareness or knowledge about the issue. In fact, one student’s medical education made them more aware of their privilege and stereotypes concerning minoritized communities (Romano, 2018). Romano (2018) discussed systems of oppression in medical professions and ended with telling readers that awareness is key to solving the problem. Second, the knowledge gained from incorporating aspects of the humanities and utilizing demographic data to “tell the story” about physical and social communities may have contributed to students’ awareness about SDOH and vulnerable populations. Many students cited poverty and mortality data in their papers and how the assignment was “eye-opening.” Students were able to put data into the form of an essay and tell a story, which can be quite compelling for students. Storytelling, through the photography essay assignment, may have allowed students to connect with certain physical or social communities. Sometimes, the storytelling component may be more memorable and impactful than data, facts, and figures (Passon, 2019). Faculty may want to keep this in mind when creating impactful assignments to learn about SDOH and their impact on different communities.

### Constructive feedback

One meaningful aspect of the assignment was the student evaluation data to help the instructor improve the assignment for future cohorts. Clear communication and listening are key characteristics related to good leadership practices. Following some of these steps may help faculty build their leadership skills in the classroom. First, it is ideal for instructors to communicate clear learning objectives, so students understand what is being asked of them as they complete the assignment. Learning objectives based on Bloom’s Taxonomy were provided to students in the project guidelines to provide structure, promote clarity, and guide student learning (Armstrong, 2010). In fact, providing learning objectives in the early stages of an assignment is promoted under the TILT framework (Winkelmes, 2014). Next, constructive feedback from students is vital to continuously improve assignments for future students. Here lies an opportunity for instructors to apply the Plan-Do-Study-Act or PDSA Framework to ensure future cohorts have high-quality instruction to improve their experience completing the assignment and promote academic success (Murray, 2018). While some student evaluation data may be more difficult to implement due to time allotted in the semester (e.g., splitting assignment into multiple assignments or having a non-graded assignment) or outliers, it is important to look for themes in student evaluation data. Last, student challenges should be noted so faculty are aware and can make necessary changes to promote student success. One student noted their limited comfort level discussing demographic data. Students being uncomfortable could suggest faculty are pushing students out of their comfort zone. It is important for healthcare students, including allied health professional students, to understand community demographic data as they work with different communities. Student challenges are an area to explore more in future cohorts. For example, one or two weeks in the semester may be dedicated to exploring demographic data more in detail, so students feel a bit more comfortable searching and discussing demographic data. Or professors may offer office hours to describe demographic data in a particular county to help students better understand how to interpret demographic data. In short, challenges are an opportunity to educate students and build their skillset regarding the assignment and course at hand. Collecting student evaluation data, listening to feedback, and acting upon students’ suggestions demonstrates responsibility and accountability on the part of the instructor. Regardless of our title, faculty must be committed to listening and learning, even from students, to be an effective classroom leader.

### Next steps

Faculty interested in recreating a SDOH photo essay may have students (1) only focus on structural SDOH in their photos, (2) require students to discuss health outcomes other than morbidity, and (3) add a small group discussion about the project as students are drafting their paper. Faculty may also incorporate a service-learning project with the paper, so students have an opportunity to volunteer in the community they captured their photo in. Are there creative ways in photography to capture more upstream factors and discuss their impact on health outcomes and health disparities in various physical and social communities? Another suggestion is to have students take a photo of one structural SDOH and one intermediary SDOH. The fourth and final suggestion for the assignment is to have students provide at least ten questions they may ask their patients from that community to better understand their social needs as they relate to SDOH and provide a rationale for each of those ten questions. Increasingly, we are seeing where researchers and clinicians are collecting SDOH or social needs data to better serve their patient populations (Page-Reeves et al., 2016). Adding the question component to the essay may help health professional students to critically think about how to better measure SDOH in the healthcare setting.

## Limitations

Students may be hesitant to provide criticism about the assignment if they think it will impact their grade. Faculty should be clear that constructive feedback is simply used for evaluation purposes and will not affect the student’s grade. The majority of students in our program are allied health professionals, but the assignment can easily be adapted and utilized for various healthcare programs at the undergraduate or graduate level to get a better sense of SDOH in various communities that their patients live and work in.

## Conclusions

Utilizing a SDOH photo essay to teach about the impact and interconnectedness of SDOH is feasible in an online program for healthcare professionals. Assignments like this one can promote awareness and empathy for various social and physical communities, including vulnerable populations like the homeless, low income, and racialized minorities. Last, encouraging students to provide lessons learned and constructive criticism in their assignment without negative repercussions empowers students to communicate knowledge gained, success, and challenges that help faculty members improve assignments for future cohorts.

## Supplementary information

Project Guidelines and Grading Rubric

## Data Availability

The datasets generated during and/or analyzed during the current study are not publicly available.

## Footnotes

1 Includes numbers and percentages for all non-missing data.

2 Captures the number of times health outcomes were discussed when linking social determinants of health to various health outcomes. These numbers do not count the number of students.
