# Supplementary material for: Using adult learning characteristics and the humanities to teach undergraduate healthcare students about social determinants of health
Source: Humanit Soc Sci Commun. 2023 Mar 18;10(1):114. doi: 10.1057/s41599-023-01599-w (PMC10024284; doi:10.1057/s41599-023-01599-w)
Supplement: Supplementary file 1 — Project Guidelines and Grading Rubric [file 41599_2023_1599_MOESM1_ESM.docx]

**Due Date:** Insert Due Date and Time

**Purpose:** The purpose of this assignment is to assess students’ understanding of social determinants of health (SDOH) in a community setting and assess how students interpret the impact of SDOH on health.

**Skills^[[1]](#footnote-1)^:** The SDOH photo essay will help students practice the following skills that are essential to your success in this program and professional career beyond the HCS program:

1. Knowledge – Define and identify what are considered SDOH
2. Comprehension – Identify and describe SDOH in a physical community setting; Describe community demographic data (e.g., education attainment, employment, income, etc.)
3. Application – Apply the SDOH Framework (structural vs. intermediary SDOH)
4. Analysis - Explain how SDOH affect various health outcomes (mortality, morbidity, functional limitation, health care costs, etc.)
5. Synthesis – Propose an approach to address SDOH to improve health equity
6. Evaluate – Assess the photography essay assignment

**Knowledge:** The assignment will also help students become familiar with the following important content knowledge:

1. Scholarly research
2. Community demographic data
3. Academic and scientific writing
4. SDOH and health inequities that impact individuals and communities

**Tasks:** Students should complete the following items:

1. Utilize course material (PPT slides, readings, videos, data sources, etc.), credible websites, research journal articles, etc. to complete the essay.
2. Use the American Psychological Association (APA) 7^th^ edition style, including title page, abstract, reference page, and appendices. Review APA resources, especially sample APA student paper
3. Follow photography etiquette^[[2]](#footnote-2)^ and local and state guidelines regarding COVID-19.^[[3]](#footnote-3)^
4. Take one photo of SDOH in a physical community setting and place it in the appendix of the paper. If students want to include additional pictures, those should be in the appendix of the paper also.
5. Write a 4-5-page photography essay (use the format on page 2 to structure the essay). Page count does not include title page, abstract, reference page, or appendix.
6. Use project guidelines ***and*** the grading rubric to evaluate your work before submission
7. Seek help from the instructor, if needed
8. Submit the final paper as a Word document in [insert learning management system] by the due date

Students should label the sections headers in the paper the same as the bolded headers below, and the following information should be listed in each section:

**Social Determinants of Health (SDOH)**

1. Define SDOH
2. Describe the image in your photograph and why it’s considered a SDOH
3. Explain if the image is considered a structural SDOH or intermediary SDOH

**Community Demographics**

1. Identify and describe at least **three** relevant community demographics (race/ethnicity, income, education, etc.) where the picture was taken using at least **one** of the following datasets. It may help students to compare their community to the US averages or other areas using the US Census Bureau website.^[[4]](#footnote-4)^
   1. County Health Rankings
   2. US Census Bureau

**Health Outcome**

1. Explain how the SDOH pictured in the photograph can impact at least **two** of the following health outcomes (students may discuss several health outcomes, but only two clearly defined health outcomes are required):
   1. Mortality (death)
   2. Morbidity (diseases)
   3. Life expectancy (how long an individual lives)
   4. Health care expenditures (how much someone pays in care)
   5. Health status (how a person rates their health)
   6. Functional limitations (ability to perform usual daily activities like bathing, eating, working, etc.)
2. Provide credible data or research to support the connection between the SDOH photographed and the health outcomes chosen

**Health Equity^[[5]](#footnote-5)^**

1. Describe one, feasible approach to improve health equity regarding your chosen SDOH

**Lessons Learned**

1. Describe “lessons learned” from completing the Photography Essay assignment
2. Recommend approaches to improve assignment for future cohorts

**Appendix**

1. Insert at least one picture of SDOH that you discuss in the photography essay.

| **Section**  **(100 points possible)** | **Excellent**  **100 (A)^[[6]](#footnote-6)^** | **Good**  **89 (B)** | **Satisfactory**  **79 (C)** | **Poor**  **69 (D)** | **Unacceptable**  **≤ 60 (F)** |
| --- | --- | --- | --- | --- | --- |
| **Writing Style /**  **APA Format**  (10 points) | 10  Four-five-page clear overview of social determinants of health (SDOH); Student clearly follows project guidelines; One photograph included in Appendix; Minor APA format/style or grammatical errors | 8  Four-five-page overview of SDOH; Student follows project guidelines; One photograph included in Appendix; Few APA format/style or grammatical errors | 7  Photography Essay is not four-five-pages; Organizational structure is vague; No photograph is included; Few APA format/style or grammatical errors | 6  Photography Essay is not four-five-pages; Organizational structure is unclear; No photograph is included; Major APA format/style or grammatical errors | 0  Needs extensive work; No attempt made |
| **Social Determinants of Health**  (20 points) | 20  Student clearly defines SDOH, clearly describes SDOH in picture; clearly discusses why or how image is SDOH; clearly states if SDOH is structural or intermediary | 18  Student defines SDOH; describes SDOH in picture; discusses why or how image is SDOH; states is SDOH is structural or intermediary | 16  Vague definition of SDOH or description of picture; Vague explanation of why image is SDOH or if SDOH is structural or intermediary | 14  Section is of poor quality; Definition of SDOH is unclear or barely noticeable; Description of picture is unclear; Unclear or barely noticeable discussion on intermediary vs. structural SDOH | 0  Needs extensive work; No attempt made |
| **Community Demographics**  (30 points) | 30  Student clearly identifies and clearly describes three relevant community demographics using at least one prescribed dataset | 27  Student identifies and describes three relevant community demographics using at least one prescribed dataset | 25  Identification of community demographics is vague; Student provides vague description of community demographics; Unclear if prescribed dataset utilized | 23  Section is of poor quality; Identification and description of community characteristics is unclear or barely noticeable; Neither of the prescribed datasets were not utilized | 0  Needs extensive work; No attempt made |
| **Heath Outcome**  (25 points) | 25  Student clearly explains how the SDOH captured in the image can impact at least two health outcomes; Student provides clear credible data or research to support the connection between SDOH and health outcomes | 23  Student explains how the SDOH captured in the image can impact at least two health outcomes; Student provides credible data or research to support the connection between SDOH and health outcomes | 20  Explanation of the relationship between SDOH and health outcomes is vague; Unclear if student utilized credible data or research to support connection between SDOH and health outcomes | 18  Section is of poor quality; Explanation of the relationship between SDOH and outcome is unclear or unnoticeable; Credible data is either unclear or barely noticeable | 0  Needs extensive work; No attempt made |
| **Health Equity**  (10 points) | 10  Student clearly describes one, feasible approach or plan to improve health equity regarding the SDOH in their photograph | 9  Student describes one, feasible approach or plan to improve health equity regarding the SDOH in their photograph | 8  Approach or plan to improve health equity is vague | 6  Section is of poor quality; Approach or plan to improve health equity is unclear or barely noticeable | 0  Needs extensive work; No attempt made |
| **Lessons Learned**  (5 points) | 5  Student clearly describes “lessons learned” from completing this assignment; Student clearly recommends approaches to improve the assignment for future cohorts | 4  Student describes “lessons” learned from completing this assignment; Student recommends approaches to improve the assignment for future cohorts | 3  “Lessons learned” and recommendations are vague | 2  Section is of poor quality; “Lessons learned” and recommendations are unclear or barely noticeable | 0  Needs extensive work; No attempt made |

**Using Adult Learning Characteristics and the Humanities to Teach Undergraduate Healthcare Students about Social Determinants of Health: Data and Lessons Learned from Photography Essays**

Elizabeth A. Brown, PhD, MPA^1,2^; Hannah Kinder^2^; Garrett Stang^2^; Wendy Shumpert^2^

^1^Old Dominion University, Norfolk, VA, United States

^2^Medical University of South Carolina (MUSC), Charleston, SC, United States

**Correspondence:** Elizabeth A. Brown, PhD; School of Community & Environmental Health; College of Health Sciences; Old Dominion University, Norfolk, VA 23529; Tel: (757) 683-6180; Email: eabrown@odu.edu

1. Skills listed follow Bloom’s taxonomy, which is a framework describing educational goals for this assignment. More information is available here: <https://cft.vanderbilt.edu/guides-sub-pages/blooms-taxonomy/> [↑](#footnote-ref-1)
2. Photography etiquette includes respecting the privacy of people and places. [↑](#footnote-ref-2)
3. Different towns, cities, counties, and states have varying policies regarding mask mandates. Please adhere to those in your local area. Further, students should (1) wear an appropriate face mask, (2) practice physical distancing, and (3) avoid personal contact. [↑](#footnote-ref-3)
4. Students may use the following US Census Bureau website to check national or local demographic data: <https://www.census.gov/quickfacts/fact/table/US/PST045221> [↑](#footnote-ref-4)
5. Health equity means everyone has a fair and equitable chance to be as healthy as possible. Health equity is different from health equality. More information about health equity is here: <https://www.rwjf.org/en/library/research/2017/05/what-is-health-equity-.html> [↑](#footnote-ref-5)
6. Clarity includes writing clear sentences/thoughts that support understanding and utilizing credible evidence to support claims. [↑](#footnote-ref-6)
